# Global Genotype-Phenotype Correlations in *Pseudomonas aeruginosa*

**DOI:** 10.1371/journal.ppat.1001074

**Published:** 2010-08-26

**Authors:** Claudia Pommerenke, Mathias Müsken, Tanja Becker, Andreas Dötsch, Frank Klawonn, Susanne Häussler

**Affiliations:** 1 Chronic Pseudomonas Infections, Helmholtz Center for Infection Research, Braunschweig, Germany; 2 Cellular Proteomics, Helmholtz Center for Infection Research, Braunschweig, Germany; Institute of Applied Informatics, University of Applied Sciences, Wolfenbüttel, Germany; 3 Twincore, Center for Experimental and Clinical Infection Research, a joint venture of the Helmholtz Center for Infection Research Braunschweig and the Medical School Hannover, Hannover, Germany; Massachusetts General Hospital, Harvard Medical School, United States of America

## Abstract

Once the genome sequence of an organism is obtained, attention turns from identifying genes to understanding their function, their organization and control of metabolic pathways and networks that determine its physiology. Recent technical advances in acquiring genome-wide data have led to substantial progress in identifying gene functions. However, we still do not know the function of a large number of genes and, even when a gene product has been assigned to a functional class, we cannot normally predict its contribution to the phenotypic behaviour of the cell or organism - the phenome. In this study, we assessed bacterial growth parameters of 4030 non-redundant PA14 transposon mutants in the pathogenic bacterium *Pseudomonas aeruginosa*. The genome-wide simultaneous analysis of 119 distinct growth-related phenotypes uncovered a comprehensive phenome and provided evidence that most genotypes are not phenotypically isolated but rather define specific complex phenotypic clusters of genotypes. Since phenotypic overlap was demonstrated to reflect the relatedness of genotypes on a global scale, knowledge of an organism's phenome might significantly contribute to the advancement of functional genomics.

## Author Summary

Phenotypic description has long been used to discriminate between bacteria and, with the publication of *Bergey's Manual of Determinative Bacteriology* in 1923, microbiologists began to systematically describe and define bacterial species based on lists of phenotypes. Because growth phenotypes are directly and indirectly involved in fundamental aspects of bacterial physiology and evolution, and remain a cornerstone of microbial taxonomy, we decided to systematically analyze the Harvard *Pseudomonas aeruginosa* non redundant PA14 transposon mutant library for a large set of different phenotypes (119) and to describe a genome wide growth-related phenotypic landscape. Our aim was to explore whether we can define subgroups of *P. aeruginosa* mutants exhibiting distinct patterns of complex trait phenotypes and to forge a link between genotype and phenotype.

## Introduction

A principal goal of genomics is to acquire a global overview of the genetic information of a cell, its bioinformatic decoding into cellular metabolism in the context of the prevailing environment, and the expression of this metabolism as cellular-organismal phenotypes. Though much progress has been made in linking genomics and metabolism, including the application of systems approaches, involving modeling and computational analyses [1], [2], the link with phenotypes remains tenuous [3].

Once the genome sequence of an organism is obtained, attention turns from identifying genes to understanding their function, their organization and control of metabolic pathways and networks that determine its physiology [4], [5]. The classical approach to define the function of individual and related groups of genes is to analyze mutant phenotypes in a systematic manner [6]–[8]. The scrutiny of comprehensive mutant libraries under a wide range of experimental conditions has revealed many new phenotypic traits and assigned them to cognate genes [9]–[11].

Most microbes exhibit only a few physically-recognizable phenotypes, limited largely to cell shape and colony morphology when cultured on solid media, but a wealth of metabolic phenotypes, many of which can be scored as color reactions involving dye-linked substrates incorporated into the culture medium. Phenotypic description has long been used to discriminate between bacteria and, with the publication of *Bergey's Manual of Determinative Bacteriology* in 1923 [12], microbiologists began to systematically describe and define bacterial species based on lists of phenotypes. Because growth phenotypes are directly and indirectly involved in fundamental aspects of bacterial physiology and evolution, they remain a cornerstone of microbial taxonomy. Here, we present a new approach to forge a link between genotype and phenotype using *Pseudomonas aeruginosa*, a bacterium exhibiting a complex lifestyle, on one hand, as a clinically problematic, antibiotic resistant facultative pathogen causing a variety of life-threatening infections of e.g. the cystic fibrosis lung, burn wounds, eye and ear, urinary tract, and on the other, as an environmental microbe ubiquitously present in soil and water. *P. aeruginosa* was one of the first human pathogens whose genome was sequenced [13], and transposon mutant libraries of two strains, PA14 and PAO1, have been generated and made available to the research community [14], [15].

In this study, we have systematically analyzed the non-redundant PA14 mutant library [15] for a large set of different phenotypes in order to develop a genome-wide, growth-related phenotypic landscape, the phenome.

## Results/Discussion

In order to define the phenome of PA14, we assessed a collection of 136 bacterial growth parameters of the comprehensive non-redundant PA14 transposon mutant library [15]. This library harbors 5776 transposon mutants with transposon insertions in 4433 unique genes with one PA14 locus identifier. In order to maximize the genome coverage, we concentrated phenotypic profiling on these 4433 single gene transposon mutants. Substrate-specific growth curves were measured in the semi-automated VITEK2 system (bioMérieux), which distinguishes bacterial species on the basis of 48 distinct biochemical reactions; the same system was also used to examine antibiotic resistance profiles based on growth curves on medium containing different concentrations of 19 antibiotics in clinical use (detailed information on the specific tests is provided in Table S1). Further growth-related phenotypic tests included the morphology of colonies on sheep's blood agar plates after 48h of growth, and the potential of all PA14 mutants to form biofilms. Phenotypic data collection, processing and analysis are described in the Material and Method section. Due to poor growth of some transposon mutants and due to incorrect measurements by the VITEK semi-automated system, we failed to acquire exploitable VITEK data of 403 mutants. Still, for 4030 (91%) of the mutants of the PA14 NR transposon mutant library the phenotypic profile was complete and thus used as the basis for the determination of the *P. aeruginosa* PA14 phenome.

### Determination of the robustness and discriminating potential of the various phenotypic tests

**Figure 1:**
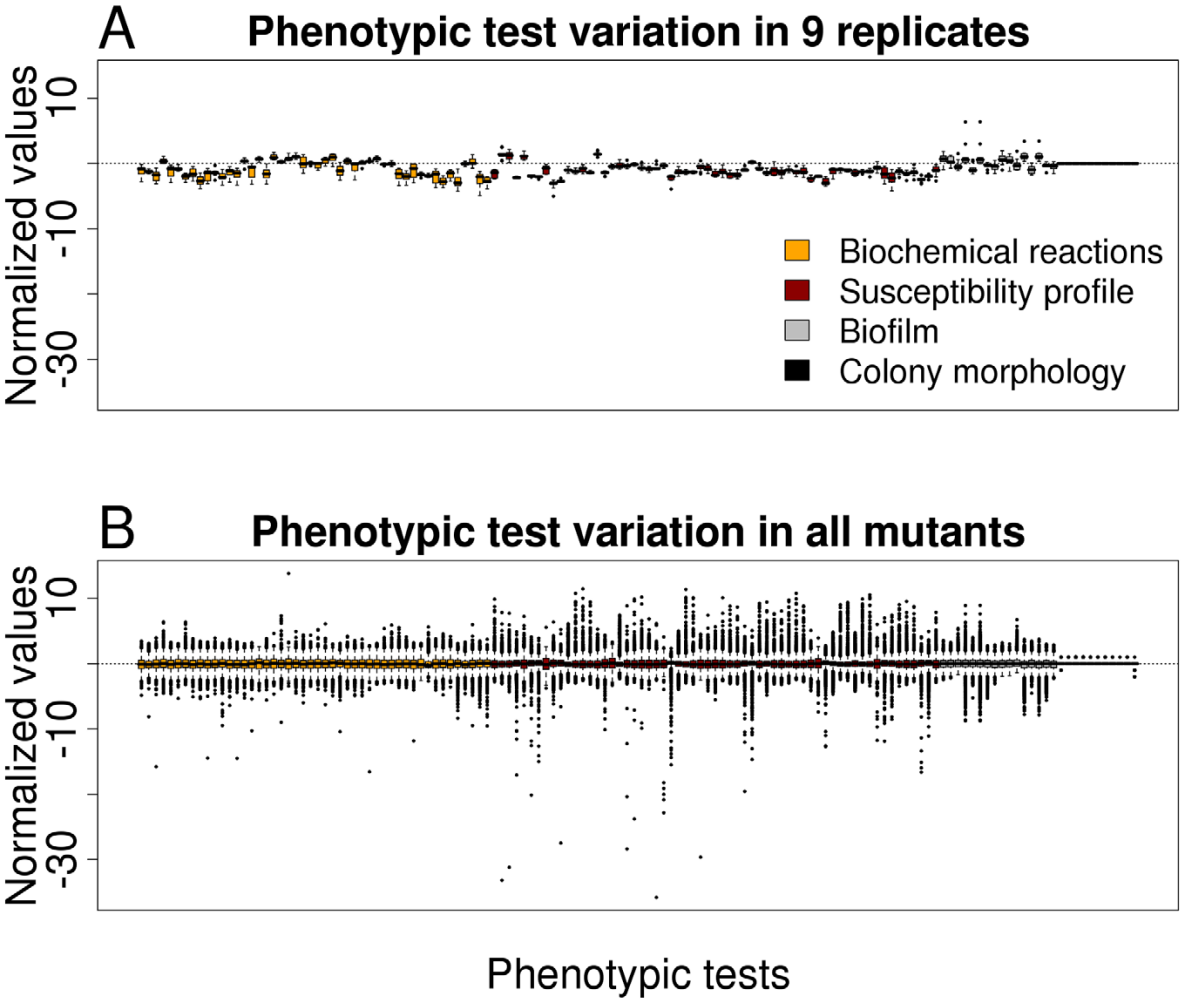
Variation in phenotypic tests. (A) Boxplot representation for preprocessed phenotypic data of nine replicates (PA14 wild-type harboring a spontaneous mutation within *flgF*) for all 136 phenotypic tests. (B) Boxplot representation of preprocessed phenotypic data of all mutants for all 136 phenotypic tests. The low variation of the replicates indicates the robustness of the testing system, whereas the high variation in all mutants reflects the discriminative potential for each test.

In order to get an impression on the robustness and the discriminating potential of the various phenotypic tests, nine replicates of the PA14 strain (harboring a spontaneous mutation within the fglF gene) were tested and the results were compared with that of all mutants. Value distributions after data importation by Java procedures and subsequent analysis by R routines for each phenotypic test are depicted in Figure 1. Because the PA14 strain used for replica determination was not a PA14 wild-type, the values as depicted in Figure 1A deviate from the horizontal centre line. A low variation within the replicates indicate the robustness of the testing system, whereas a high variation within all mutants (Figure 1B) reflect the discriminative potential for each test. With the aim to exclude those phenotype tests that firstly, did not reveal robust and reproducible data and secondly, had only a low discriminative power (e.g. testing of the aminoglycoside resistance profile, since all transposon mutants harbored a gentamycin cassette), we compared the replicate variances with the variances of all transposon mutant data. Those phenotypic tests whose replicate variances exceeded those of all mutants as well as those without evident difference between the variances were excluded from further analysis (F-test, one-sided, p>0.5). Of 136 applied tests 119 validated phenotypic tests remained for further analysis, of 48 biochemical tests 38 were included in the study and of 61 antibiotic tests 59, respectively. (The phenotypic tests that were excluded are indicated in Table S1.)

### Distinct phenotpyic patterns are revealed by hierarchical clustering

**Figure 2:**
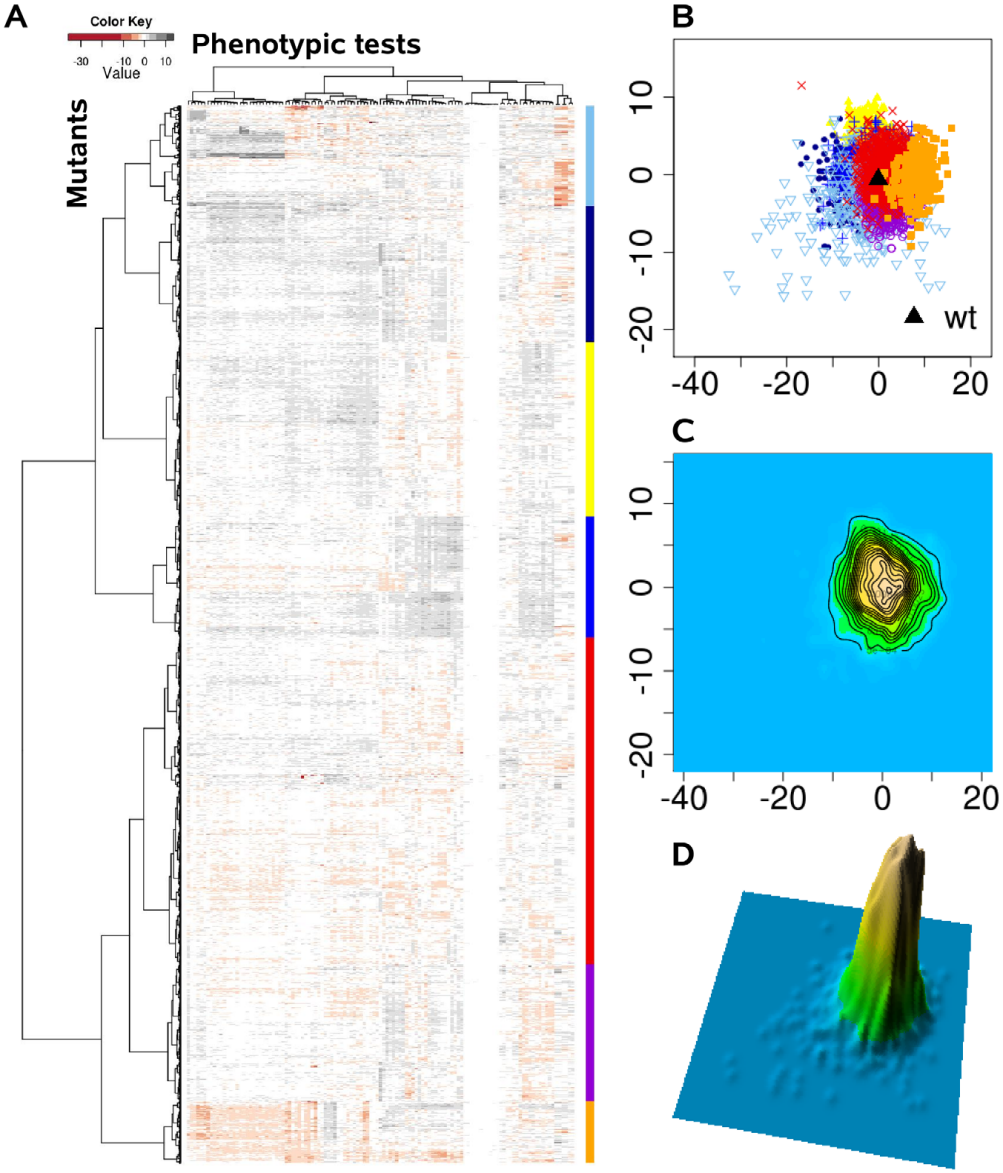
The *P. aeruginosa* phenome landscape organized by phenotypic similarity determined by 119 phenotypes of 4030 mutants. We systematically estimated the phenotypic distances between the genotypes, which were the basis for hierarchical clustering and multi-dimensional scaling. (A) Heat map, rows correspond to mutants and columns to different phenotypic tests. (B) Projection onto a two-dimensional grid using multi-dimensional scaling (MDS). Point colors were overlaid by seven cluster groups. (C) MDS data were converted to a contour image using two-dimensional kernel density estimation. Altitudes indicate the density of mutants in the MDS grid. (D) The contour image was depicted as a three-dimensional landscape, in which phenotypically similar mutants are located close to each other, the height indicating the density of mutants in that region of the phenotypic space.

**Figure 3:**
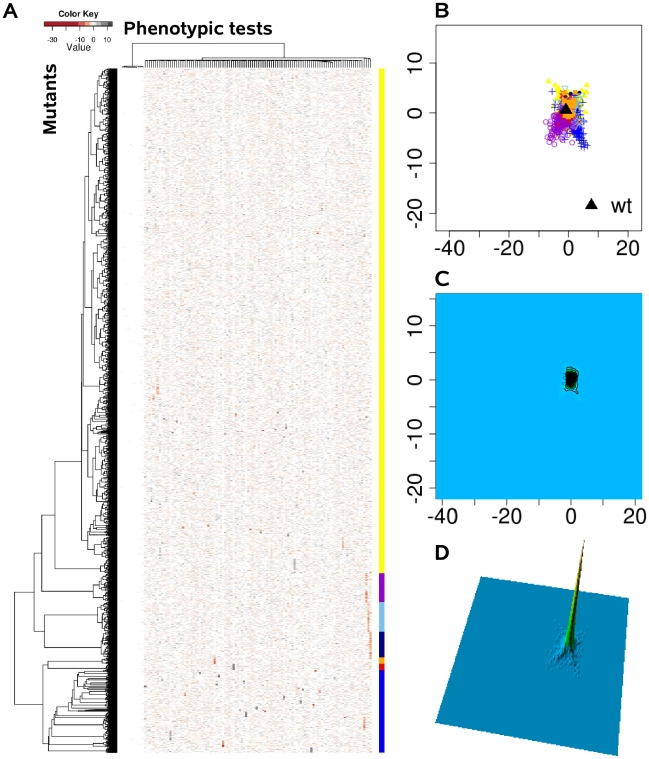
The phenome landscape for permutated values. Phenotypic distances between randomly shuffled values served as the basis for hierarchical clustering and multi-dimensional scaling. (A) Heat map, rows correspond to mutants and columns to different phenotype test. (B) Projection onto a two-dimensional grid using multi-dimensional scaling (MDS). Point colors were overlaid by seven cluster groups. (C) MDS was converted to a contour image using two-dimensional kernel density estimation. Altitudes indicate the density of mutants in the MDS grid. (D) The contour image was depicted as a three-dimensional landscape. In comparison to Figure 2 the different cluster group symbols spread homogeneously and in smaller space.

Instead of viewing phenotypes as independent entities, we applied a comprehensive statistical and visualization method to identify clusters of complex trait phenotypes exhibiting distinguishable patterns and to ultimately discern new relationships between genes, proteins and cellular pathways. In doing so, subgroups of overlapping complex traits phenotypes might evolve as more information becomes available, and dynamically merge or split as further information is acquired. Based on the assumption that each of the applied 119 single phenotypic tests is equally capable of detecting disturbances within the cellular network, we calculated the phenotypic distance between the genotypes as Euclidean distance in a 119 dimensional space. Although the condensation of 119 dimensions of the different phenotypic tests simplifies the data available and overestimates many tests, clear clustering patterns were extracted from the complex phenotypic traits, as visualized in the heat map (Figure 2A). These clusters were absent, if the phenotypic test results were permutated among the 4030 mutants (Figure 3A). The relatedness of the mutant phenotypes also became apparent on a plane via multidimensional scaling (Figure 2B) and visualization as a contour map (Figure 2C) and a three-dimensional landscape by kernel density estimation (Figure 2D). Notably, phenome mappings revealed that the majority of mutant phenotypes cluster around the wild-type phenotype, which is consistent with the notion that most mutants behave similar to the wild-type and differ in only few specific tests. However, it also becomes obvious that most mutants are not phenotypically isolated but rather form a part of a continuum of related phenotypic traits.

### Global network of phenotypic relatedness

Two common challenges arise with analyzing high-dimensional data [16]–[18]. One is called the curse of dimensionality that for any point the Euclidean distance between its closest neighbor and the farthest point diminishes with increasing dimensionality. The other challenge is based upon the phenomenon that the complexity of many existing data mining algorithms grows exponentially with increasing dimensionality. Consequently, the relatedness of phenotypes determined here by a simple hierarchical cluster analysis of Euclidean distances may be strongly underestimated.

Concentrating on the discriminative phenotypic profiles of the mutants, we determined the Jaccard index (JI) [19] of similarly discriminative phenotypic tests of individual gene pairs (a Jaccard index of 0/1 indicates no/complete agreement, respectively). A mutant was assigned to exhibit a discriminative phenotype for a specific test, if the value for the individual mutant was found outside two standard deviation of the median value of all mutants. Under these conditions the wild-type showed no discriminative phenotypic tests and over 70% of the mutants behaved differently in less than 10% of the tests and 93% of the 4030 mutants showed a change in at least one phenotypic trait. This is comparable to what was shown before in the analysis of a chemical portrait of yeast. By applying 1144 chemical genomic assays Hillenmeyer *et al.* [11] observed a measurable growth phenotype for 97% of yeast gene deletions.

**Figure 4:**
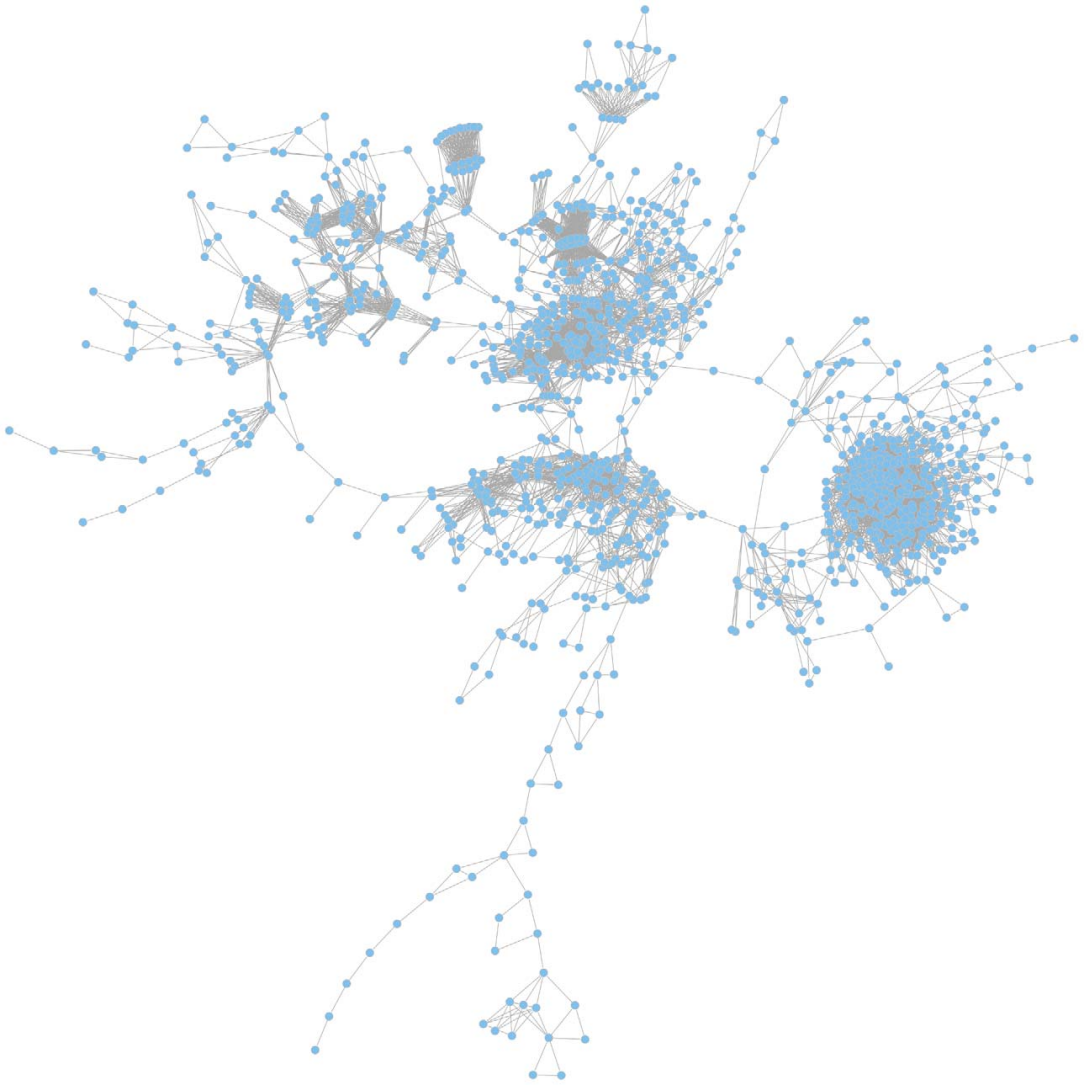
The global phenotypic network. The network is based on calculated Jaccard indices (JIs), a measure for gene relatedness. In the largest coherent graph 1196 genes are represented by the nodes and the connections between the genes are drawn for gene pairs with JI>0.51. A zoomable graph with gene names is provided in the supporting information (Figure S1).

Based on the calculated JIs we constructed a global coherent network which visualizes relatedness of the mutant phenotypes on a global scale. In such a network, only those mutant phenotypes are depicted which exhibit a phenotype that differs from the wild-type and that is shared with other mutant phenotypes (Figure 4).

### Functional gene relations are reflected in the global network

In order to determine whether the 119 phenotypic tests we have applied are sufficient for defining complex trait phenotypes that are shared by genetically homogeneous subsets of mutants, we next focused our analysis on specific phenotypic fingerprints that characterize gene pairs known to be functionally related, and compared their relatedness with those of the phenotypic patterns of randomly selected pairs. Functionally related gene pairs were assigned on the basis of either their organization within an operon [20], gene ontology (GO) or a common gene name designation [21], thus capturing the genomic context as well as broader functional relatedness.

Most interestingly, the level of similarity (i.e. JI) between genes that were described to be functionally related proved to be significantly higher than that of non-related genes. The JIs of all gene pairs except the replicates were included in this analysis ((4029-1)*4029/2 = 8114406). Among those JIs, 2856 gene pairs exhibited a common gene name, 1493 were located within one operon, and 10011 grouped into the same gene ontology term. The JIs of all those three groups proved to be significantly different to the JIs of the remaining non-related gene pairs (common gene name: p<2.2E-16, operon: p = 2.0E-4, GO: p<2.2E-16, Mann-Whitney-U test, one-sided) [22].

Of note, there are confounding constrains on our analysis namely assignment of about 3% of the transposon mutants are expected to be false, there are potential of polar effects in the transposon mutant library, and yet unknown gene connections contribute to the randomly chosen non-related gene pairs, which would even underestimate the true difference between functional related and non-related genotypes.

Taken together, our finding indicates that the described phenotypic landscape is indeed able to detect functional relationships on a global scale. The phenotype network might reveal new relationships between genes, proteins and cellular pathways that are not displayed by sequence based functional categorization and might even be used for the functional classification of hypothetical genes, which still make up more than 40% of the *P. aeruginosa* genome [23]. Furthermore, vice versa the knowledge of a distinct phenotypic pattern of a *P. aeruginosa* strain might be used to predict its genomic make-up and a specific phenotypic pattern which is exhibited following perturbation might be used to uncover cellular pathways affected by the respective stress.

### Conclusion

In order to fully exploit the power of correlating genotypes to phenotypes, we did not only analyze discrete phenotypic entities on a genome-wide scale but instead searched for common overlapping phenotypic traits that link complex mutant phenotypes on the basis of key shared features. Our data provide a proof of principle that phenotypic overlap can result from genes that specify similar functions and attest that an organism's phenome might predict relatedness of genotypes on a functional scale. The phenome landscape might not only be a novel tool for gene assignment, but the knowledge of a distinct phenotypic pattern of a *P. aeruginosa* isolate might furthermore be used to predict its genomic make-up and the expression of a distinct phenotypic pattern might also uncover the affection of cellular pathways as a result of a specific perturbation, a tool which would significantly advance e.g. the search for the mode of action of novel drugs.

## Materials and Methods

### Phenotype data generation and processing

The automated VITEK2 system records antibiotic susceptibility based on bacterial growth curves and metabolic substrate conversions/consumptions over time. The ID-GN card was used for the evaluation of biochemical reactions and the AST-N063 card for antimicrobial susceptibility testing [24] (Table S2). Each of the 4433 mutants from the Harvard PA14 non-redundant mutant library [15] was measured once except for the PA14 strain (harboring a spontaneous mutation within the *flgF* gene) which was measured nine times. The curves received for the strains were processed in several steps: (a) Since the time intervals for the recording of the transmission values by the VITEK system varied substantially (5-28min.), we adjusted them to a constant time interval of 15 min. The values for the specific time points (5, 20, 35 min., etc.) were calculated by linear interpolation. (b) The VITEK measurements were discarded when i) the initial transmission values differed considerably (as determined by visual inspection) from that of the majority of all tested mutants and ii) when the initial transmission values did not show the typical increase due to foil warming and a gradual dissolution processes within the first 100 minutes of the measurement. (c) If the measurements of one individual card for one transposon mutant failed for at least 10 tests, the results of the card were declared non-reliable and this mutant was excluded from further analysis (overall 403 mutants were discarded). (d) For the antibiotic tests, curves with antibiotic treatment were subtracted from the control curves without treatment. (e) Median polish for each specific time point was applied on the data assuming an additive-fit model [25]. (f) The VITEK curves were condensed to one median value of the polished transmission values over time for one mutant and one single test. (g) Continuous data were subjected to median-interquartile range normalization.

*P. aeruginosa* biofilms of all mutants were grown on the bottom of 96-well plates for 48h, stained with the LIVE/DEAD *Bac*Light Bacterial Viability Kit (Molecular Probes/Invitrogen) and monitored by the use of an automated confocal laser scanning microscope (Opera system) as described previously [26]. Confocal microscopy biofilm grey scale 8/16 bit bmp-images were prepared via Auto PHLIP-ML [27] to black/white 8 bit images and further processed via the open source MatLab toolbox PHLIP [28] resulting in 16 different PHLIP parameters for each measured mutant. These included 8 parameters each for red and green fluorescence. Continuous data were subjected to median-interquartile range normalization for each parameter.

11 distinct colony morphology characteristics were identified as different/similar (1/0) to the wild-type colony morphology by visual inspection and recorded in the phenotypic profiles matrix. Data analyses and visualization were conducted in the R environment (www.r-project.org).

### Landscape visualization

In order to visualize the phenome landscape of *P. aeruginosa* the multidimensional phenotypic profile of each mutant needed to be reduced to a two-dimensional distance matrix. From the phenotypic fingerprint matrix of n by k, the n by n distance matrix (lower triangle) was computed using the Euclidean distance, n being the number of genes and k the number of phenotype tests. Secondly, in order to visualize the gene connections as topographical landscapes, multidimensional scaling was used for placing genes on the plane. The reliefs of the landscapes were estimated by two dimensional kernel density. In the third step, genes were allocated to seven specific cluster groups identified by the hierarchical clustering (Ward's method) and labeled in distinct colors and symbols (Figure 2, Figure 3). The R package gplots provided useful functions for clustering and coloring.

### Assigning functionally related genes

For assigning functionally related genes, we checked for their organization within an operon, or a common gene name designation. Hereby, the genomic context as well as broader functional relatedness were captured. Short common gene names were assigned as provided for the non-redundant *Pseudomonas aeruginosa* PA14 Transposon Insertion Mutant Library (http://ausubellab.mgh.harvard.edu/cgi-bin/pa14/downloads.cgi), *Pseudomonas* Genome Database v2 [21]. In case of multiple gene name designation the gene name provided by the *Pseudomonas* Genome Database was given priority. Operons were retrieved from the DOOR database [20] and GO terms from the *Pseudomonas* Genome Database [21]. Operons or common gene names with at least 2 members were used for further analysis (Table S2).

### Validation of gene correlation via the Jaccard Index

We determined the distribution of the data for all mutants for each phenotypic test. An individual mutant was defined as exhibiting a discriminative phenotype for a specific test, if its value was found outside two standard deviation of the median value of all mutants (two-sided). For normally distributed data approximately 95% values are located within two standard deviations of the mean. The standard deviation was estimated via the interquartile range of the original values. The Jaccard indices (JI, [19]) for the sets of discriminating tests of two given genes were calculated as a measure for phenotypic similarity between the genes:
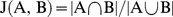
with A as the set of discriminating phenotypic test for gene A and B as the set for gene b.

For determining significant differences of JI of related gene pairs the Mann-Whitney-U test was applied [22].

### Global network visualization

We constructed a global coherent network which visualizes relatedness of the mutant phenotypes on a global scale based on Jaccard indices. We visualized our findings as a graph with genes being the nodes and their pair-wise relations as edges. Connections were selected for JI>0.51, for which the ratio TPR/FPR was maximal, and the largest coherent graph was depicted only. Graphs were spread according to the Kamada Kawai algorithm [29]. The R packages igraph and Rgraphviz proved to be useful in this context.

## Supporting Information

Figure S1 Global network. A detailed zoomable graphic of the networks shown in Figure 4. Gene names are provided.
(0.80 MB PDF)

Table S1 List of applied phenotypic tests. Phenotypic traits that were selected for the definition of the PA14 phenome are indicated.
(0.16 MB DOC)

Table S2 List of selected common gene names and operons. For the common gene name designation 317 gene groups were found with at least two genes. The first three letters of the gene short names are listed. 485 operon groups were provided by the DOOR DB with at least two genes for which one was specified here.
(0.03 MB DOC)
