# Supplementary material for: Global Genotype-Phenotype Correlations in Pseudomonas aeruginosa
Source: PLoS Pathog. 2010 Aug 26;6(8):e1001074. doi: 10.1371/journal.ppat.1001074 (PMC2928780; doi:10.1371/journal.ppat.1001074)
Supplement: Table S1 — List of applied phenotypic tests. Phenotypic traits that were selected for the definition of the PA14 phenome are indicated. (0.16 MB DOC) [file ppat.1001074.s002.doc]

Table S1: Phenotypic traits measured and selected for further analysis as indicated.

| **Test type** | **Phenotypic trait** | **Wavelength [nm] / Concentration [µg/mL] / Color fluorescence** | **Selected tests** |
| --- | --- | --- | --- |
| **VITEK GN card** | beta-galactosidase | 430 |  |
|  | beta-N-acetyl-glucosaminidase | 430 | x |
|  | glutamyl-arylamidase-pNAl | 430 |  |
|  | gamma-glutamyl-transferase | 430 | x |
|  | beta-glucosidase | 430 |  |
|  | beta-xylosidase | 430 | x |
|  | beta-alanine-arylamidase-pNA | 430 | x |
|  | alpha-glucosidase | 430 |  |
|  | beta-N-acetyl-galactosaminidase | 430 |  |
|  | alpha-galactosidase | 430 |  |
|  | phosphatase | 430 | x |
|  | glycine-arylamidase | 430 | x |
|  | beta-glucuronidase | 430 | x |
|  | glu-gly-arg-arylamidase | 430 |  |
|  | Ellman | 430 | x |
|  | ala-phe-pro-arylamidase | 660 |  |
|  | adonitol | 660 | x |
|  | L-pyrrolydonyl-arylamidase | 660 |  |
|  | L-arabitol | 660 | x |
|  | D-cellobiose | 660 | x |
|  | H2S-production | 660 | x |
|  | D-glucose | 660 | x |
|  | fermentation/glucose | 660 |  |
|  | D-maltose | 660 | x |
|  | D-mannitol | 660 | x |
|  | D-mannose | 660 | x |
|  | L-proline-arylamidase | 660 | x |
|  | lipase | 660 |  |
|  | palatinose | 660 | x |
|  | tyrosine-arylamidase | 660 |  |
|  | urease | 660 | x |
|  | D-sorbitol | 660 | x |
|  | saccharose/sucrose | 660 | x |
|  | D-tagatose | 660 | x |
|  | D-trehalose | 660 | x |
|  | citrate (sodium) | 660 |  |
|  | malonate | 660 | x |
|  | 5-keto-D-gluconate | 660 |  |
|  | L-lactate-alkalinisation | 660 | x |
|  | succinate-alkalinisation | 660 | x |
|  | ornithine-decarboxylase | 660 |  |
|  | lysine-decarboxylase | 660 | x |
|  | L-histidine-assimilation | 660 |  |
|  | coumarate | 660 | x |
|  | O/129-resistance (comp.vibrio.) | 660 | x |
|  | L-malate-assimilation | 660 |  |
|  | L-lactate-assimilation | 660 | x |
| **VITEK AST-N063** | positive control | 0 | x |
|  | Ampicillin | 4 | x |
|  | Ampicillin | 8 | x |
|  | Ampicillin | 32 | x |
|  | Ampicillin/Sulbactam | 4 / 2 | x |
|  | Ampicillin/Sulbactam | 16 / 8 | x |
|  | Ampicillin/Sulbactam | 32 / 16 | x |
|  | Cefazolin | 4 | x |
|  | Cefazolin | 16 | x |
|  | Cefazolin | 64 | x |
|  | Cefepime | 2 | x |
|  | Cefepime | 8 | x |
|  | Cefepime | 16 | x |
|  | Cefepime | 32 | x |
|  | Cefotaxime | 1 | x |
|  | Cefotaxime | 4 | x |
|  | Cefotaxime | 16 | x |
|  | Cefotaxime | 32 | x |
|  | Cefoxitin | 8 | x |
|  | Cefoxitin | 16 | x |
|  | Cefoxitin | 32 | x |
|  | Cefpodoxime | 0.5 | x |
|  | Cefpodoxime | 1 | x |
|  | Cefpodoxime | 4 | x |
|  | Ceftazidime | 1 | x |
|  | Ceftazidime | 2 | x |
|  | Ceftazidime | 8 | x |
|  | Ceftazidime | 32 | x |
|  | Cefuroxime | 2 | x |
|  | Cefuroxime | 8 | x |
|  | Cefuroxime | 32 | x |
|  | Ciprofloxacin | 0.5 | x |
|  | Ciprofloxacin | 2 | x |
|  | Ciprofloxacin | 4 | x |
|  | Ertapenem | 0.5 | x |
|  | Ertapenem | 1 | x |
|  | Ertapenem | 6 | x |
|  | Gentamicin | 4 |  |
|  | Gentamicin | 16 |  |
|  | Gentamicin | 32 |  |
|  | Levofloxacin | 0.5 | x |
|  | Levofloxacin | 4 | x |
|  | Levofloxacin | 8 | x |
|  | Meropenem | 0.5 | x |
|  | Meropenem | 4 | x |
|  | Meropenem | 16 | x |
|  | Nalidixic Acid | 8 | x |
|  | Nalidixic Acid | 16 | x |
|  | Nalidixic Acid | 32 | x |
|  | Piperacillin | 4 | x |
|  | Piperacillin | 16 | x |
|  | Piperacillin | 64 | x |
|  | Piperacillin/Tazobactam | 4 / 4 | x |
|  | Piperacillin/Tazobactam | 16 / 4 | x |
|  | Piperacillin/Tazobactam | 128 / 4 | x |
|  | Tigecycline | 0.75 | x |
|  | Tigecycline | 2 |  |
|  | Tigecycline | 4 |  |
|  | Tobramycin | 8 | x |
|  | Tobramycin | 16 | x |
|  | Tobramycin | 64 | x |
|  | Trimethoprim/Sulfamethoxazole | 0.5 / 9.5 | x |
|  | Trimethoprim/Sulfamethoxazole | 2 / 38 | x |
|  | Trimethoprim/Sulfamethoxazole | 16 / 304 | x |
| **Biofilm** | Total biovolume [µm3] | green |  |
|  | Substratum coverage [%] | green |  |
|  | Surface area to biovolume ratio [µm-1] | green | x |
|  | Horizontal spreading [µm2] | green | x |
|  | Vertical spreading [µm2] | green |  |
|  | Total spreading [µm2] | green | x |
|  | Mean thickness [µm] | green | x |
|  | Biofilm roughness | green |  |
|  | Total biovolume [µm3] | red |  |
|  | Substratum coverage [%] | red |  |
|  | Surface area to biovolume ratio [µm-1] | red | x |
|  | Horizontal spreading [µm2] | red | x |
|  | Vertical spreading [µm2] | red |  |
|  | Total spreading [µm2] | red | x |
|  | Mean thickness [µm] | red | x |
|  | Biofilm roughness | red |  |
| **Morphology** | color |  | x |
|  | transparency |  | x |
|  | convex |  | x |
|  | circular |  | x |
|  | smooth |  | x |
|  | crumbly |  | x |
|  | opaque |  | x |
|  | slimy |  | x |
|  | shiny |  | x |
|  | autolysis |  | x |
|  | size |  | x |
