# Supplementary material for: Global Genotype-Phenotype Correlations in Pseudomonas aeruginosa
Source: PLoS Pathog. 2010 Aug 26;6(8):e1001074. doi: 10.1371/journal.ppat.1001074 (PMC2928780; doi:10.1371/journal.ppat.1001074)
Supplement: Table S2 — List of selected common gene names and operons. For the common gene name designation 317 gene groups were found with at least two genes. The first three letters of the gene short names are listed. 485 operon groups were provided by the DOOR DB with at least two genes for which one was specified here. (0.03 MB DOC) [file ppat.1001074.s003.doc]

**Table S2: List of selected common gene names and operons.**

For the common gene name designation 317 gene groups were found with at least two genes. The first three letters of the gene short names are listed. 485 operon groups were provided by the DOOR DB with at least two genes for which one was specified here.

| **Common gene names** | **Operon, via DOOR DB** |
| --- | --- |
| acc, ace, aco, acs, adh, agu, ahp, ald, alg, alk, ami, amp, amr, ans, ant, aot, apa, apb, aph, apr, arc, arg, arn, aro, ars, aru, asn, asp, atp, ats, ben, bet, bfr, bio, bkd, bra, btu, cad, car, cat, cbr, ccm, cco, che, chp, cio, cit, clp, cob, cod, cox, crc, cre, csp, csu, ctp, cup, cya, cyc, cyn, cyo, cys, czc, dac, dad, dap, dct, ddl, din, dip, dna, dpp, dsb, emr, est, eut, exa, exb, exo, exs, fab, fad, fdh, fdn, fec, fep, fim, fix, fkl, fle, flg, flh, fli, foa, fol, fpt, fpv, fru, fts, fum, gab, gap, gbu, gcd, gcv, glc, glg, glm, gln, glo, glp, glt, gny, gpF, gsh, has, hcn, hcp, hem, hfl, his, hit, hpa, hpc, hpl, hsl, htp, hut, hxc, hyi, ilv, isc, kat, kdp, kgu, lep, leu, lip, lld, lpd, lpx, lys, map, mdc, met, mex, mgt, mia, mlt, mms, mnt, moa, mod, mot, mox, msu, mtl, muc, mut, nad, nag, nap, nar, nas, nir, nor, nos, nqr, ntr, nud, nuo, nus, omp, opd, opm, opr, orf, ORF, osm, paa, pan, par, pbp, pca, pch, pcr, pct, pdh, pdx, pel, pep, pfe, pfp, pha, phh, phn, pho, phu, phz, pil, pir, piu, plc, pls, pmr, pnc, pob, pop, pot, ppk, pqq, pqs, prm, pro, prp, psc, psl, pst, pur, put, pvc, pvd, pyk, pyr, qor, rad, rar, rbs, rcs, rec, rhl, rim, RL1, rlu, rnf, rpm, rpo, rps, RS0, RS1, rsm, rub, ruv, sbc, sco, sda, sec, sel, ser, sfn, slt, sox, spe, spu, ssb, ssp, ssu, suc, sug, thi, thr, tnp, top, tox, trk, trm, trp, tru, trx, ubi, ure, uvr, vac, van, wbp, wsp, wzm, xcp, xdh, xer, xyl, yad, yae, yaf, yai, ybd, ybe, ybi, yca, ycg, yci, ycj, ydg, yea, yec, yed, yeg, yfc, yfd, yfj, ygf, ygi, ygj, yhd, yhh, yhi, yic, yie, yig, yjc, yji, yli, yrb, znu, zwf | trkA, PA14_21150, PA14_40600, PA14_55020, plcB, PA14_21580, PA14_40650, PA14_55200, PA14_00340, PA14_21650, PA14_40700, PA14_55250, trpA, PA14_21710, PA14_40730, PA14_55590, ycgB, PA14_21870, aphA, sbcD, glpE, PA14_21900, nudC, PA14_55780, pdxA, vacJ, PA14_41010, PA14_55850, PA14_07780, yjcH, PA14_41110, PA14_56030, PA14_07890, bapA, clpP, PA14_56090, gph, yrbF, PA14_41260, PA14_56210, PA14_08000, ykgJ, nasS, PA14_56260, gpW, yciV, nasA, PA14_56450, gpFI, gltR, nirB, PA14_56510, PA14_08180, gltF, cmpX, PA14_56560, PA14_08260, zwf, PA14_41690, PA14_56620, PA14_08310, hutG, PA14_41740, PA14_56750, trpG, yciK, PA14_41800, PA14_56800, PA14_08520, serC, PA14_42030, PA14_56840, phzE1, ORF_10, PA14_42140, PA14_56880, opmD, PA14_23520, pscL, inaA, PA14_09680, kdgA, exsB, PA14_57140, dapA, ygfF, popD, PA14_57480, PA14_09770, nqrA, pscR, sspB, PA14_09930, PA14_25410, PA14_42670, PA14_57650, fepG, pilZ, PA14_42750, cysN, acoC, PA14_25820, stk1, ttg2D, cvaB, PA14_26090, PA14_43030, yrbH, hpcC, hisM, PA14_43090, rpoN, PA14_10960, PA14_26300, ybbB, PA14_57990, PA14_11030, btuC, kdpB, PA14_58080, cupB1, cobH, PA14_43420, gatA, PA14_11210, PA14_26540, PA14_43480, PA14_58230, PA14_11280, PA14_26600, PA14_43520, PA14_58300, PA14_11480, PA14_26650, PA14_43660, PA14_58350, PA14_11590, moxR, PA14_43780, PA14_58410, PA14_11630, PA14_27140, PA14_43840, PA14_58530, PA14_11700, PA14_27400, PA14_43870, piuC, PA14_11740, PA14_27490, sucD, ampE, eutC, PA14_27690, yijF, pilC, PA14_11920, yadG, PA14_44190, PA14_58820, PA14_11960, PA14_27800, cytN, PA14_58890, proA, PA14_27940, fixG, RL114, PA14_12300, PA14_28100, xdhA, RL108, thiE, PA14_28560, PA14_44840, PA14_59130, PA14_12440, PA14_28580, PA14_44900, PA14_59200, PA14_12590, PA14_28820, ttuD, pilL2, PA14_12690, PA14_28920, PA14_45070, PA14_59480, PA14_12840, PA14_29150, PA14_45120, PA14_59520, PA14_12940, tetV, PA14_45170, PA14_59560, cioA, PA14_29240, PA14_45210, PA14_59620, PA14_13130, yaiW, PA14_45260, PA14_59640, PA14_13330, PA14_29340, PA14_45520, cupD4, PA14_13350, wzm, cheZ, rcsB, PA14_13590, fhp, flhF, PA14_59820, PA14_13670, PA14_29680, PA14_45700, PA14_59860, narK2, PA14_29710, PA14_45840, PA14_59980, PA14_13870, nuoN, PA14_45870, PA14_60070, PA14_14000, PA14_30030, PA14_45920, PA14_60100, PA14_14140, PA14_30070, PA14_46000, pilS, PA14_14370, PA14_30130, gbuA, pilW, PA14_14480, PA14_30210, potF, PA14_60410, queA, ftsK, PA14_46510, PA14_60570, PA14_14690, PA14_30470, PA14_46570, PA14_60630, PA14_14710, PA14_30520, PA14_46750, fdhD, PA14_14830, PA14_30590, PA14_46810, attL, ybdL, gacA, PA14_46850, PA14_68780, PA14_15160, PA14_30850, gltJ, gcvH1, PA14_15260, PA14_30990, ansB, PA14_68890, PA14_15590, PA14_31060, PA14_47120, PA14_68970, PA14_15620, PA14_31110, cyoE, yhhJ, nagE, PA14_31190, PA14_47250, ubiB, yfjD, PA14_31270, phnX, ppk, trmD, PA14_31350, PA14_47320, PA14_69330, hom, PA14_31680, PA14_47360, hemY, PA14_16210, PA14_31720, ycgL, algR, PA14_16290, PA14_31870, PA14_50820, PA14_69520, wspA, cztS, PA14_50890, PA14_69580, PA14_16560, xylX, PA14_51040, cyaA, PA14_16630, antC, opdD, lysA, PA14_16720, PA14_32330, purC, PA14_70010, PA14_16880, PA14_32360, perM, PA14_70050, PA14_16960, oprN, phnB, PA14_70480, dapC, PA14_32440, pqsE, PA14_70530, yaeL, PA14_32480, spcU, PA14_70560, adhC, dipZ2, RS05, rubB, surE, PA14_32640, PA14_51640, glcF, PA14_17550, PA14_32830, PA14_51690, ubiC, PA14_17590, PA14_32860, ruvB, phoB, potD, PA14_32930, PA14_51980, pstB, ycjJ, PA14_33120, PA14_52080, betA, PA14_17810, PA14_33190, relA, PA14_71000, glpK, PA14_33310, pirS, PA14_71120, ybaK, PA14_33340, lemA, PA14_71190, mmsA, PA14_33380, PA14_52350, PA14_71250, PA14_18160, PA14_33460, aruE, ltaA, fruB, PA14_33530, argR, glyA1, PA14_18300, PA14_33570, dctM, PA14_71580, algJ, PA14_33610, PA14_52870, PA14_71670, algX, pvdO, phhA, PA14_71690, PA14_18600, opmQ, PA14_53090, zwf, mexE, aldG, PA14_53120, PA14_71840, PA14_18810, PA14_33970, PA14_53160, uvrD, nth, PA14_34110, bolA, wbpZ, PA14_19030, msuE, fumC2, PA14_72010, yigM, metN-1, PA14_53310, PA14_72080, PA14_19210, mtlZ, plcH, prfH, PA14_19350, PA14_34460, PA14_53400, PA14_72230, ssuA, tauD, tdcD, algB, ssuD, mxaA, PA14_53650, PA14_72420, nosD, PA14_35010, PA14_53770, PA14_72500, PA14_20260, arsB, PA14_53860, PA14_72520, phnD, kguT, PA14_53920, np20, amiE, pvcD, yecO, abc, PA14_20620, lpdV, rnc, PA14_72650, PA14_20640, PA14_39990, mucD, dctD, PA14_20680, nuoA, opdH, PA14_72750, PA14_20700, PA14_40230, PA14_54600, PA14_72790, PA14_20770, xqhA, PA14_54910, PA14_72850, PA14_20960, PA14_40350, PA14_54950, PA14_73010, PA14_21040, modA, PA14_54970, PA14_73050, PA14_73100, PA14_73200, atpG, parB |
