# Supplementary material for: Global Genotype-Phenotype Correlations in Pseudomonas aeruginosa
Source: PLoS Pathog. 2010 Aug 26;6(8):e1001074. doi: 10.1371/journal.ppat.1001074 (PMC2928780; doi:10.1371/journal.ppat.1001074)

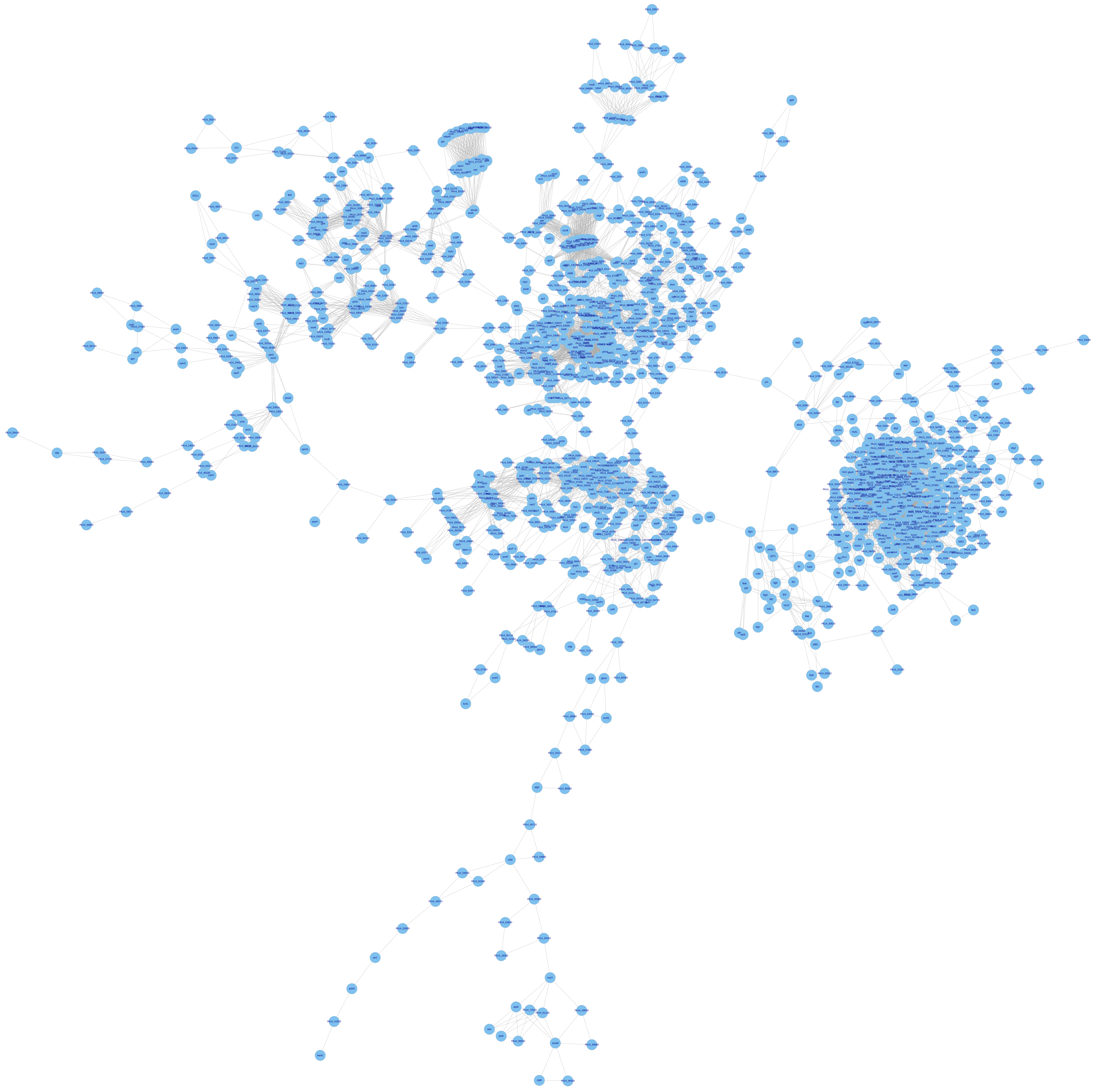

Supplement: Figure S1 — Global network. A detailed zoomable graphic of the networks shown in Figure 4. Gene names are provided. (0.80 MB PDF) [file ppat.1001074.s001.pdf]
